# Artificial intelligence-guided clavicle fracture detection on plain radiographs: A retrospective diagnostic accuracy study

**DOI:** 10.1097/MD.0000000000049488

**Published:** 2026-06-26

**Authors:** Nikolai Ramadanov, Robert Hable, Maximilian Voss, Roland Becker, Andreas G. Schreyer, Robert Prill, Mikhail Salzmann

**Affiliations:** a Center of Orthopaedics and Traumatology, University Hospital Brandenburg, Brandenburg/Havel, Germany; b Faculty of Health Science Brandenburg, Brandenburg Medical School Theodor Fontane, Brandenburg/Havel, Germany; c Faculty of Applied Computer Science, Deggendorf Institute of Technology, Deggendorf, Germany; d Department of Diagnostic and Interventional Radiology, University Hospital Brandenburg, Brandenburg/Havel, Germany.

**Keywords:** artificial intelligence, clavicle fracture, diagnostic accuracy, fracture detection, machine learning, orthopedic surgery, radiography

## Abstract

Extensive research exists on artificial intelligence (AI)-assisted fracture detection in various anatomical regions, but clavicle fractures remain comparatively understudied. This study aimed to compare the diagnostic performance of AI-guided clavicle fracture detection on plain radiographs with that of an experienced orthopedic surgeon. We retrospectively analyzed all clavicle radiographs obtained at our institution between September 11,2023 and October 31,2024 following the clinical implementation of AI-guided fracture detection. Radiological findings confirmed by a senior radiologist served as the reference standard. AI performance was additionally compared with the assessments of an experienced, blinded orthopedic surgeon. Diagnostic performance metrics included accuracy, sensitivity, specificity, Cohen kappa, F1 score, and Youden index. A total of 367 clavicle radiographs were included, comprising 186 anteroposterior (AP) and 181 second-plane views. The mean patient age was 44.6 ± 26.2 years, and 59.4% were male. According to the reference standard, clavicle fractures were present in 96 AP radiographs (51.6%) and 95 second-plane radiographs (52.5%). On AP radiographs, the AI system achieved an accuracy of 97.31%, sensitivity of 95.83%, specificity of 98.89%, Cohen kappa of 0.95, and F1 score of 0.97. Diagnostic performance on second-plane radiographs was similarly high and overall comparable to that of the experienced orthopedic surgeon. This retrospective diagnostic accuracy study shows that AI-guided clavicle fracture detection is highly accurate and reliable, with performance approaching that of an experienced orthopedic surgeon. These findings support the potential role of AI as a diagnostic support tool in clavicle fracture detection, while prospective and multicenter validation remains warranted.

## 1. Introduction

Clavicle fractures are among the most common injuries in orthopedic and trauma care, accounting for approximately 3 to 10% of all fractures.^[1]^ They are particularly prevalent in children and young adults, often resulting from falls onto the lateral shoulder or direct trauma.^[1]^ Accurate and timely diagnosis is essential to prevent complications such as malunion or neurovascular damage. While clinical evaluation is crucial, radiographs remain the standard imaging modality for confirming clavicle fractures and guiding treatment decisions.^[2]^ In certain cases, particularly with complex or subtle fractures, computed tomography (CT) scans may be employed to provide detailed assessment.^[3]^

Historically, radiologists have been relied upon to interpret medical images such as radiographs and CT scans to identify fractures.^[4]^ However, recent advances in artificial intelligence (AI) have led to the development of automated systems designed to assist or even replace physicians in detecting fractures. AI-based fracture detection methods leverage advanced technologies, particularly deep learning algorithms, to analyze large datasets of medical images with remarkable speed and accuracy. These systems have demonstrated the potential to reduce human error and enhance diagnostic precision by identifying subtle or complex fractures that may be missed by the human eye.^[5]^ Studies comparing AI systems to human raters have reported promising results, with some models achieving diagnostic performance on par with or surpassing that of experienced radiologists.^[6–10]^ As these technologies continue to evolve, they are being increasingly integrated into medical diagnostics, offering new possibilities for improving patient care and optimizing clinical workflows.

Despite extensive research on AI in detecting fractures across various anatomical regions, clavicle fractures remain comparatively understudied.^[11–13]^ Furthermore, existing studies often rely on AI models trained on data from a single geographic region or developed using specific technologies, potentially limiting their generalizability. Variability in patient demographics, imaging equipment, and clinical workflows across regions can significantly influence AI performance, highlighting the need for validation studies using diverse datasets and alternative AI systems. A study with a different regional focus and developer can address these variabilities, ensuring that findings are robust and applicable to a broader population.

This study aimed to compare the performance of AI in detecting clavicle fractures on plain radiographs with the performance of human raters. By addressing gaps in the literature and testing the generalizability of AI in a new context, this research contributes to advancing knowledge in AI-based fracture detection and its potential clinical applications.

## 2. Methods

This single-institution retrospective data analysis was approved by the institutional review board of Brandenburg Medical School (231072024-BO-E-RETRO), and the requirement for informed consent was waived. The waiver was granted due to the retrospective nature of the study, where data was anonymized and no direct patient interaction occurred. The institutional review board determined that the study posed minimal risk to participants and that obtaining informed consent was not feasible. All methods were performed in accordance with the relevant guidelines and regulations.

### 2.1. Study sample

We retrospectively analyzed all clavicle radiographs taken in our hospital since the introduction of AI-guided fracture detection from September 11,2023 to October 31,2024. There was no predetermined target number for radiographs; data collection was based on all eligible cases within this period that met the inclusion criteria. Inclusion criteria were as follows: patients across all age groups; who received radiographs in 1 or 2 planes of the clavicle; with adequate image quality and field of view, defined as radiographs that were clear, properly exposed, and fully captured the clavicle and surrounding anatomical structures without significant artifacts or distortion. Although the AI system is validated for patients aged ≥ 2 years, all eligible radiographs (including a small number of infants) were included to reflect real-world emergency department practice; performance in this subgroup should therefore be interpreted with caution.

Anteroposterior (AP) radiographs were obtained with the patient positioned upright, the affected shoulder closest to the radiograph cassette, and the arm in a neutral position. Second-plane radiographs, typically an axial (cephalocaudal) clavicle view, were performed to provide additional perspectives for fracture evaluation and classification. These complementary views allow for better visualization of displacement, angulation, and complex fracture patterns, thereby enhancing diagnostic accuracy.

All straight AP and second-plane radiographs were identified in our institution’s radiographic demonstration program by using predefined search criteria based on body region and examination date. The results of the AI and the responsible radiologist were then extracted into a spreadsheet. In addition, the images were assessed by an experienced orthopedic surgeon and his findings on fracture detection were also included in the spreadsheet. This spreadsheet provided the necessary data for subsequent statistical analysis. The orthopedic surgeon was blinded to study design, patient demographics and radiograph results from both the AI system and the radiologist. This ensured that no unintentional bias was introduced in the assessment process, allowing for a robust and impartial evaluation of the radiographs.

### 2.2. Radiographs assessment

The assessment of the radiographs taken was automatically evaluated by the AI system and the AI result was added to a copy of the original radiograph, which could be easily accessed by any physician involved. Each radiograph was individually assessed by an experienced, blinded radiologist. The senior radiologist was involved in confirming the findings made by the responsible radiologist, ensuring the accuracy of the reports. In accordance with routine clinical practice, the radiological report confirmed by a senior radiologist was used as the reference standard, without additional consensus adjudication or systematic CT confirmation. Accordingly, cases with discordant findings between the AI system, the orthopedic surgeon, and the reference standard were not separately adjudicated, but were retained in the analysis and categorized as false-positive or false-negative results relative to the reference standard.

### 2.3. BoneView version 2.5.1

The AI system BoneView version 2.5.1 (Gleamer) has been implemented and fully used in the clinical environment of our hospital since September 11,2023.BoneView is a commercially available AI-based diagnostic support tool. It is a Conformité Européenne (French for “European Conformity”)-marked medical device designed to detect fractures, effusions, dislocations and focal bone lesions on digital imaging and communications in medicine images. The algorithm covers analysis of the lower and upper limbs, pelvis, thoracolumbar spine and chest for patients aged 2 years and older. The underlying algorithm utilizes deep learning techniques, specifically a convolutional neural network, to analyze medical images and identify abnormalities.

**Figure 1.**
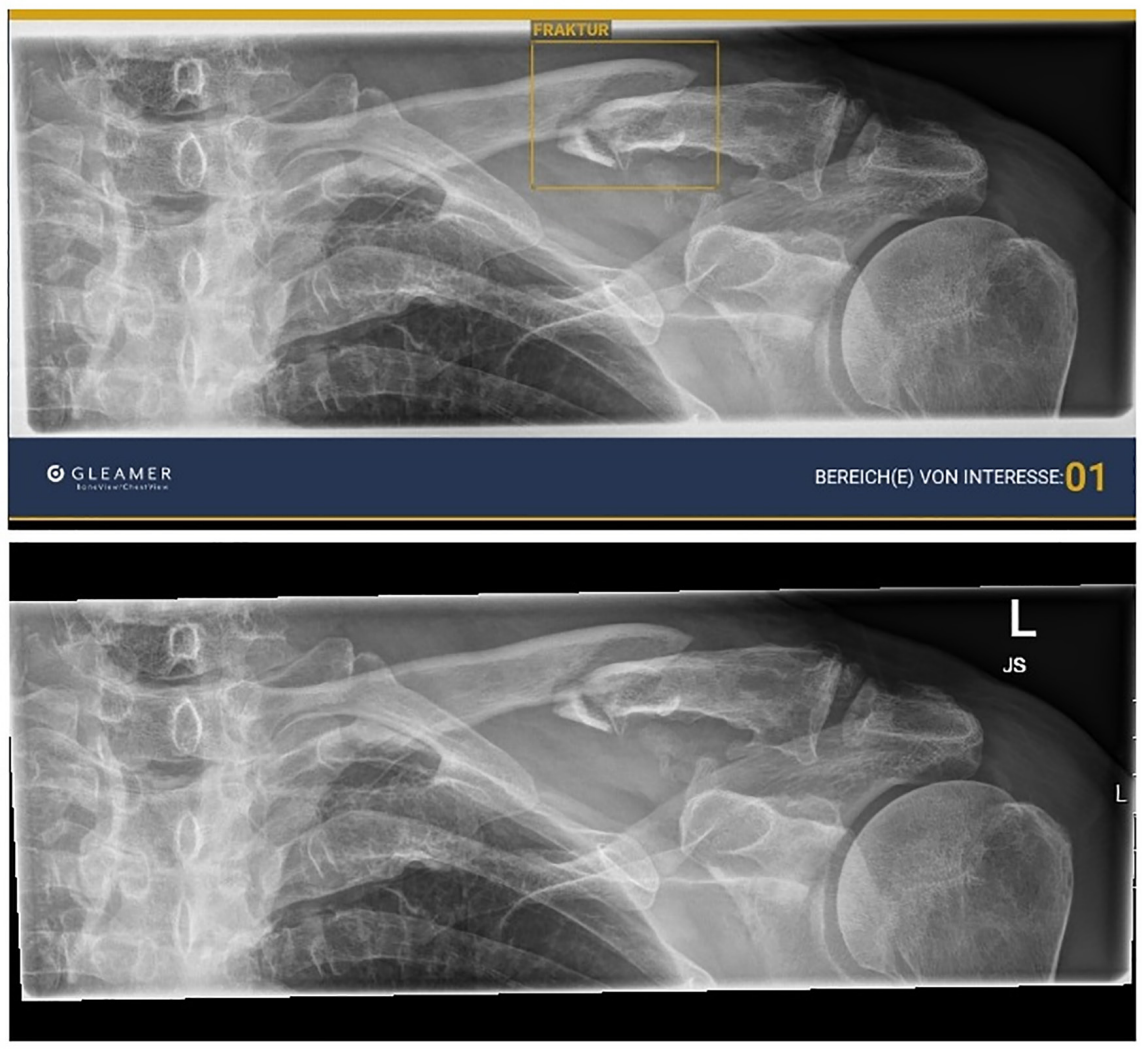
Native AP radiograph of a clavicle fracture and detection by the BoneView AI system. AP = anteroposterior.

The algorithm uses 2 operating points: “doubtful” if the confidence score is between 50% and 90%, and “positive” if the confidence score is above 90%.^[14]^ A confidence score between 0% and 50% gives a negative result.^[14]^ These operating points were chosen from the receiver operating characteristic curve after internal testing to optimize the balance between sensitivity (%) and specificity (%). For ease of use, the software highlights the region of interest by using a rectangular box on the radiographs, using a continuous line for positive results and a dotted line to indicate doubt. Figure 1 shows a native AP radiograph of a clavicle fracture and detection by the BoneView AI system (Fig. 1).

### 2.4. Statistical analysis

The following parameters were calculated to evaluate the performance of AI-guided clavicle fracture detection compared to human raters by a professional statistician using R version 4.2.1 (R Foundation for Statistical Computing, Vienna, Austria). Radiological findings were accepted as ground truth.

True Positives (TP), True Negatives (TN), False Positives (FP), and False Negatives (FN) were derived directly from the confusion matrix generated by comparing the AI model predictions to the ground truth.

Accuracy was calculated as the proportion of correctly classified cases (TP + TN) out of the total number of cases. Accuracy = (TP + TN)/ (TP + TN + FP + FN).

Cohen Kappa coefficient was used to measure inter-rater agreement beyond chance between the AI model and human raters. κ = (Po − Pe)/ (1 − Pe), where Po is the observed agreement, and Pe is the expected agreement by chance.

The F1 score was calculated as the harmonic mean of precision (positive predictive value) and sensitivity (%) (recall). F1 = 2 × ((Precision × Sensitivity)/ (Precision + Sensitivity)), where Precision = TP/ (TP + FP).

Sensitivity (Recall), or TP rate, was calculated as the proportion of actual positives correctly identified by the AI system. Sensitivity = TP/ (TP + FN).

Specificity, or TN rate, was calculated as the proportion of actual negatives correctly identified. Specificity = TN/ (TN + FP).

The Youden Index (J Statistic) was used to summarize the diagnostic effectiveness of the model. J = Sensitivity + Specificity − 1.

In addition to the primary analysis, exploratory subgroup analyses were performed to further characterize AI performanceaccording to fracture location (midshaft vs non-midshaft) and displacement status (displaced vs non-displaced). Owing to the exploratory post hoc nature of these analyses and the limited size of some subgroups, findings were interpreted descriptively and as hypothesis-generating.To compare paired binary diagnostic outcomes between the AI system and the orthopedic surgeon, McNemar test was additionally performed for AP and second-plane radiographs. A *P* value < .05 was considered statistically significant.

## 3. Results

### 3.1. Descriptive results

**Figure 2.**
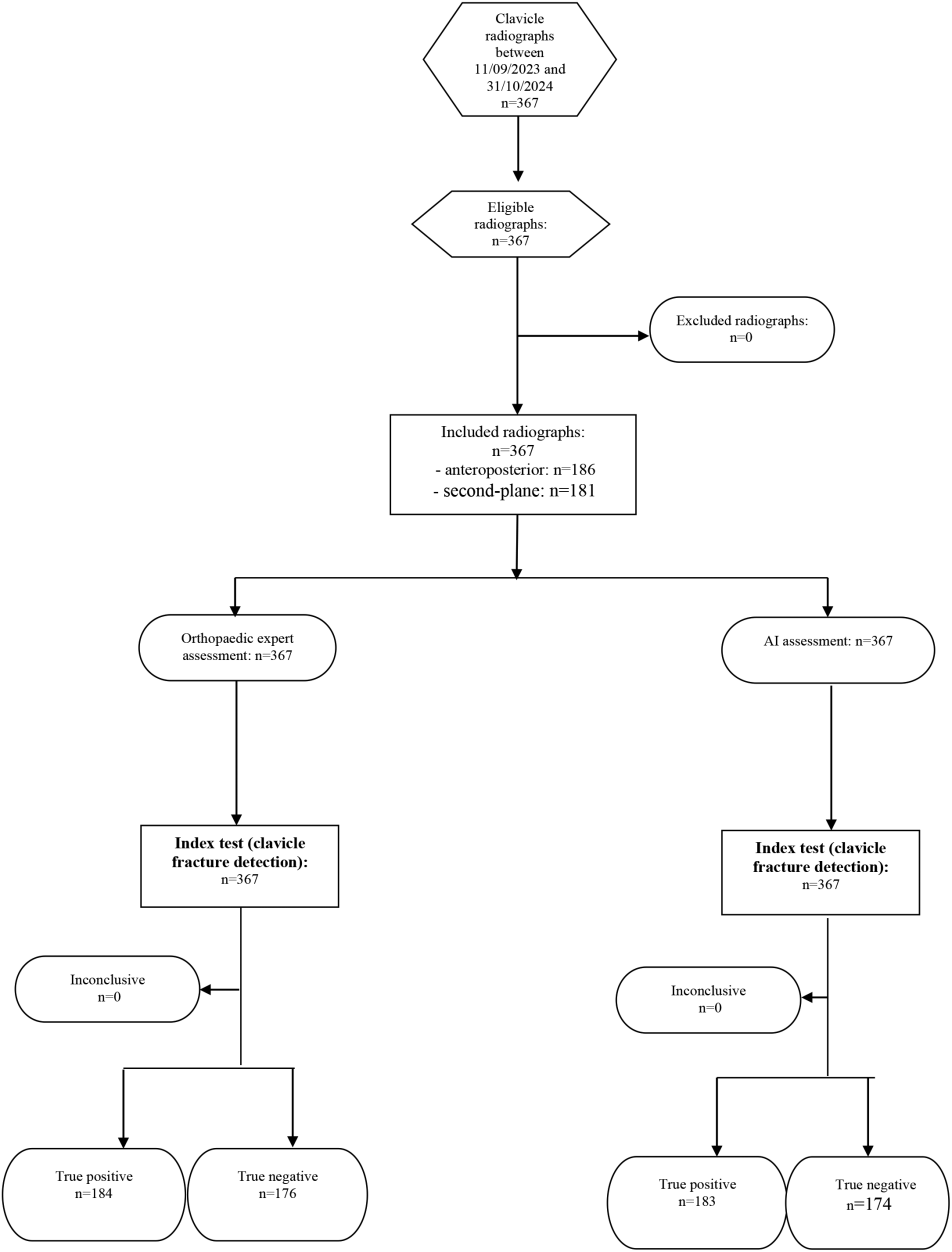
Flowchart diagram. AI = artificial intelligence, n = number of radiographs.

Between September 11, 2023, and October 31, 2024, a total of 367 plain radiographs (AP and second-plane) of the clavicle were taken at our hospital (Fig. 2). These radiographs were captured on 186 examination days and included 186 AP radiographs and 181 second-plane radiographs. The second-plane view was missing in 5 cases due to patient noncompliance or radiation safety considerations for children. These 186 radiographs were performed on 175 unique patients, with 11 patients appearing twice in the dataset (different examination dates and radiographs). The mean age of the patients was 44.6 years (± 26.2), ranging from 0 to 94 years. Of these, 59.4% were male and 40.6% were female. Clavicle fractures were identified in 51.6% of the AP radiographs (96 cases) and 52.5% of the second-plane radiographs (95 cases), based on the radiological ground truth. Table 1 provides a descriptive analysis of the included radiographs (Table 1). Clavicle fractures predominantly involved the midshaft (~78%), followed by lateral (~18%) and medial (~4%) locations, with approximately 2-thirds (~67%) being displaced.

**Table 1:** Descriptive analysis of the included radiographs.

Parameter	Value
Patients (N)	175
Sex (N, %)	Male: 104 (59.4%)
	Female: 71 (40.6%)
Age (yrs ± SD; min–max)	44.6 ± 26.2; 0.8–94.5
Total number of radiographs (N)	367
Radiographs (N) on different examination dates	186
Anteroposterior plain radiograph (N)	186
Missing:	0
Fracture (N, %)	No: 90 (48.4%)
	Yes: 96 (51.6%)
Second-plane radiograph	181
Missing	5
Fracture	No: 86 (47.5%)
	Yes: 95 (52.5%)

SD = standard deviation.

### 3.2. Clavicle fracture detection metrics

Radiological findings were considered the ground truth for all subsequent analyses.

1. Accuracy
  - AP radiographs: AI achieved an accuracy of 97.31%, while the orthopedic surgeon achieved 97.85% (Fig. 3, Table 2).
  - Second-plane radiographs: AI achieved an accuracy of 97.24%, compared to the surgeon’s accuracy of 98.34% (Fig. 3, Table 2).
2. Cohen Kappa
  - AP radiographs: AI demonstrated a Kappa value of 0.95, while the orthopedic surgeon’s Kappa was 0.96 (Fig. 4, Table 2).
  - Second-plane radiographs: AI achieved a Kappa value of 0.95, compared to the surgeon’s 0.97 (Fig. 4, Table 2).
3. F1 Score
  - AP radiographs: The F1 score for AI was 0.97, while the surgeon achieved 0.98 (Fig. 4, Table 2).
  - Second-plane radiographs: AI’s F1 score was 0.97, compared to 0.98 for the surgeon (Fig. 4, Table 2).
4. Sensitivity
  - AP radiographs: AI and the surgeon both achieved a sensitivity of 95.83% (Fig. 3, Table 2).
  - Second-plane radiographs: AI’s sensitivity was 95.79%, while the surgeon’s was 96.84% (Fig. 3, Table 2).
5. Specificity
  - AP radiographs: AI demonstrated a specificity of 98.89%, while the surgeon achieved 100% (Fig. 3, Table 2).
  - Second-plane radiographs: AI’s specificity was 98.84%, compared to 100% for the surgeon (Fig. 3, Table 2).
6. Youden Index
  - AP radiographs: The Youden Index for AI was 94.72, while the surgeon’s was 95.83 (Fig. 3, Table 2).
  - Second-plane radiographs: AI’s Youden Index was 94.63, compared to 96.84 for the surgeon (Fig. 3, Table 2).

**Table 2:** Statistical analysis of AI performance compared to human raters.

	AI on AP radiograph (95% CIs)	Orthopaedic surgeon on ap radiograph (95% CIs)	AI on second-plane radiograph (95% CIs)	Orthopaedic surgeon on second-plane radiograph (95% CIs)
Accuracy	97.31 (93.86–98.85)	97.85 (94.60–99.16)	97.24 (93.70–98.81)	98.34 (95.24–99.43)
Cohen Kappa	0.95 (0.90–0.99)	0.96 (0.92–1.00)	0.95 (0.90–0.99)	0.97 (0.93–1.00)
F1 score	0.97 (0.95–0.99)	0.98 (0.95–1.00)	0.97 (0.95–0.99)	0.98 (0.96–1.00)
Sensitivity	95.83 (89.77–98.37)	95.83 (89.77–98.37)	95.79 (89.67–98.35)	96.84 (91.12–98.92)
Specificity	98.89 (93.97–99.80)	100.00 (95.91–100.00)	98.84 (93.70–99.79)	100.00 (95.72–100.00)
Youden Index	94.72 (89.67–98.92)	95.83 (91.30–99.03)	94.63 (89.65–98.88)	96.84 (92.71–100.00)
TP	92	92	91	92
TN	89	90	85	86
FP	1	0	1	0
FN	4	4	4	3

AI = artificial intelligence, AP = anteroposterior, CI *= *confidence interval, FN = false negatives, FP = false positives, TN = true negatives, TP = true positives.

**Figure 3.** AI performance on AP and second-plane radiographs for accuracy, sensitivity, specificity and Youden Index compared to human raters. AI = artificial intelligence, AP = anteroposterior.

**Figure 4.** AI performance on AP and second-plane radiographs for Cohen Kappa and F1 score compared to human raters. AI = artificial intelligence, AP = anteroposterior.

Table 2 provides detailed metrics for both AI and the orthopedic surgeon, including accuracy, Cohen Kappa, F1 score, sensitivity, specificity, Youden Index, as well as counts of TP, TN, FP, and FN for AP and second-plane radiographs. McNemar test demonstrated no statistically significant difference between the AI system and the orthopedic surgeon for fracture detection on either AP radiographs (*P* = .20) or second-plane radiographs (*P* = .10).

### 3.3. Exploratory subgroup analysis

Exploratory subgroup analyses stratified by fracture location (midshaft vs non-midshaft) and displacement status (displaced vs non-displaced) did not reveal any meaningful differences in AI diagnostic performance across subgroups. These findings should be interpreted cautiously, as some subgroup sizes were limited and the analyses were not prespecified.

## 4. Discussion

### 4.1. Main findings

This retrospective data analysis of 367 plain radiographs demonstrates that the AI-guided clavicle fracture detection system performs with high accuracy, approaching the diagnostic capabilities of an experienced orthopedic surgeon. While both AI and the surgeon showed comparable sensitivity and agreement, the surgeon achieved slightly higher specificity. These findings highlight that the AI system is reliable and capable of supporting clinical workflows, though human expertise retains a slight advantage in precision. These comparisons should be interpreted in the context that performance of the human comparator reflects the judgments of a single experienced orthopedic surgeon. Individual diagnostic thresholds and expertise may have influenced specificity and agreement metrics; therefore, future studies incorporating multiple orthopedic readers with varying experience levels are needed to better capture interobserver variability and enhance generalizability.

### 4.2. The AI system BoneView version 2.5.1

BoneView is a commercially available AI-based diagnostic support tool that uses a convolutional neural network based on Detectron2.^[14]^ Detectron2 is an open source object detection platform developed by Facebook AI Research and implemented using PyTorch (https://pytorch.org/), an open source machine learning framework that facilitates the construction of deep learning projects.^[14]^ A dataset of 500,000 patient radiographs from 22 radiology departments between January 2011 and May 2023 was used to develop the algorithm. This dataset was then randomly divided into 80% for training, 5% for validation and 15% for internal testing. The training dataset included 20% of patients under the age of 18.^[14]^ Utilizing deep learning techniques, the system analyzes radiographs and categorizes findings into “positive,” “doubtful,” or “negative” based on optimized confidence thresholds. These technological foundations, combined with rigorous training and validation protocols, contribute to the system’s high diagnostic accuracy and reliability observed in this study. However, further studies are needed to explore its adaptability to datasets outside its training distribution.

### 4.3. Comparison with existing literature

The results of this study are consistent with existing literature on AI applications in clavicle fracture detection. Magnéli et al (2023) reported an area under the curve (AUC) of 0.96 for clavicle fractures, which is comparable to our findings.^[11]^ However, slight differences may be due to variations in dataset size, demographic characteristics, and imaging modalities. While Magnéli et al focused on shoulder radiographs of the proximal humerus, scapula and clavicle, our study concentrated only on clavicle fractures, which might contribute to differences in model performance, especially given the broader context of fracture types in our dataset.

Cheng et al (2024) evaluated an AI model for simultaneous rib and clavicle fracture detection, achieving an AUC of 0.912, with sensitivity of 86.8% and specificity of 80.4%.^[12]^ The lower AUC and specificity in their study could be attributed to the added complexity of detecting rib fractures alongside clavicle fractures in chest radiographs, while our study focused solely on clavicle fractures, which may have simplified the detection task and led to better precision in this domain.

Tsai et al (2023) applied AI to the dating of birth-related clavicular fractures in pediatric populations.^[13]^ Although this application differs from our focus on adult fractures, both studies highlight AI’s utility in detecting subtle fractures. The differences in fracture types (pediatric vs adult) and the task of dating fractures rather than simply detecting them may explain some of the variations.

Overall, the performance of our AI system is comparable to or slightly better than those in the literature, particularly in terms of specificity. Differences in results across studies are likely due to variations in datasets, fracture types, and imaging techniques, all of which reinforce the reliability of our AI system in clavicle fracture detection.

### 4.4. Implications for clinical practice

The integration of AI in radiology has profound implications for clinical practice.^[15]^ The AI system’s ability to analyze radiographs with high accuracy and agreement can reduce the workload of radiologists, particularly in high-volume emergency or trauma settings.^[16]^ Automating routine fracture detection allows radiologists to focus on more complex cases requiring nuanced interpretations. The AI’s performance metrics, particularly specificity, suggest it could minimize FP and FNs, critical in ensuring timely and accurate diagnosis.^[17]^ This is particularly relevant for subtle or occult fractures, where human error rates are traditionally higher.^[5]^ Accurate fracture detection is also clinically important because treatment strategies for clavicle fractures range from conservative management to surgical fixation depending on factors such as fracture displacement, shortening, comminution, associated injuries, and patient functional demands. Precise radiographic assessment therefore directly influences treatment planning and clinical outcomes.^[18]^ Training AI with a larger dataset of radiographs, particularly cases confirmed by magnetic resonance imaging or CT, could further improve diagnostic performance, enabling it to better tolerate radiograph errors and distinguish subtle abnormalities with higher reliability. The system’s robustness makes it an ideal tool in settings where expert radiologists are unavailable, such as rural or underserved areas. General practitioners and emergency physicians might benefit from AI as a decision-support tool to enhance diagnostic accuracy.

The utilization of AI tools has the potential to enhance the efficiency of clinical workflows by prioritizing cases that necessitate urgent attention, thereby enabling radiologists to allocate their attention to more complex analyses.^[19]^ The integration of AI with the radiological field has been shown to improve diagnostic accuracy and reduce the occurrence of missed fractures.^[20]^ AI can also contribute to the reduction of healthcare expenditures by avoiding the repetition of imaging procedures and unnecessary consultations, thereby ensuring that diagnoses are made expeditiously and with precision. The integration of AI fracture detection systems into existing healthcare infrastructures is becoming increasingly feasible, due to advancements in electronic health records and interoperability standards.^[21]^ These innovations hold great promise in enhancing patient care, reducing diagnostic delays, and optimizing resource allocation in clinical settings.

While the AI system demonstrates high accuracy and sensitivity in detecting clavicle fractures, slight differences in specificity and F1 scores compared to expert human raters highlight some performance gaps. These discrepancies may be due to the AI’s reliance on pixel-based patterns, whereas human raters take into account contextual factors such as clinical history or patient demographics. For example, radiologists may adjust their interpretation based on the mechanism of injury or subtle signs suggesting associated trauma, which the AI currently lacks. In addition, variations in radiographic quality or artifacts can present challenges to the AI system that experienced human graders can overcome through visual reasoning.

### 4.5. Limitations and future directions

Despite the strong diagnostic performance observed in this study, several limitations should be considered when interpreting the findings. First, the reference standard was based on routine radiological reports confirmed by a senior radiologist rather than systematic cross-sectional imaging or adjudication by an independent consensus panel. While this reflects real-world clinical practice, it may have led to misclassification of subtle, non-displaced, or radiographically occult fractures and may therefore have resulted in a modest overestimation of diagnostic performance for both the AI system and the human comparator. Second, this was a single-center retrospective study conducted within 1 institutional imaging environment using 1 commercially available AI tool. As a result, the findings may not be directly generalizable to institutions with different patient populations, fracture prevalence, imaging protocols, equipment vendors, or reporting workflows. Performance estimates obtained under these conditions should therefore be interpreted as setting-specific rather than universally transferable.

Third, the human comparison was limited to 1 experienced orthopedic surgeon. Although this provides a clinically relevant benchmark, it does not capture the variability that would be expected across readers with different levels of training, subspecialty background, or diagnostic thresholds. Accordingly, the present results should not be interpreted as reflecting overall human reader performance in routine practice. Fourth, the AI system evaluated only image data and had no access to clinical information such as trauma mechanism, point tenderness, deformity, or prior imaging, all of which may influence fracture detection in equivocal cases. This likely places the AI system at a structural disadvantage in some scenarios, but also highlights a key limitation of current image-only deployment models.

Additional limitations include the small proportion of missing second-plane radiographs, the repeated inclusion of a limited number of patients at different time points, and the inclusion of a very small number of patients younger than the validated age range of the software. Although these issues are unlikely to have materially altered the overall conclusions, they further support cautious interpretation of the reported performance metrics. Importantly, high retrospective diagnostic accuracy alone should not be equated with immediate clinical readiness. Before broader implementation can be recommended, prospective multicenter studies are needed to evaluate robustness across heterogeneous clinical environments, assess integration into real-world diagnostic workflows, and determine whether AI assistance translates into fewer missed fractures, faster reporting, and clinically meaningful patient benefit. Exploratory subgroup analyses did not identify meaningful differences in AI performance according to fracture location or displacement status. However, these analyses were likely underpowered due to the limited size of some subgroups and should therefore not be interpreted as evidence of equivalence across fracture patterns. Larger studies are needed to determine whether specific fracture characteristics are associated with differential AI performance.

## 5. Conclusion

The study demonstrates that AI-guided clavicle fracture detection is highly accurate and reliable, approaching the performance of an experienced orthopedic surgeon in this single-center retrospective cohort. These findings support the potential role of AI as a diagnostic support tool in clinical practice, particularly in augmenting human capabilities in fracture detection. However, careful consideration of its limitations and validation in diverse settings are essential for widespread implementation.

## Author contributions

**Conceptualization:** Nikolai Ramadanov.

**Data curation:** Nikolai Ramadanov, Maximilian Voss.

**Formal analysis:** Nikolai Ramadanov, Robert Hable.

**Investigation:** Nikolai Ramadanov, Robert Hable.

**Methodology:** Nikolai Ramadanov, Robert Hable.

**Project administration:** Nikolai Ramadanov, Robert Hable.

**Resources:** Nikolai Ramadanov.

**Software:** Nikolai Ramadanov, Robert Hable.

**Supervision:** Nikolai Ramadanov, Robert Hable.

**Validation:** Nikolai Ramadanov.

**Visualization:** Nikolai Ramadanov.

**Writing – original draft:** Nikolai Ramadanov, Maximilian Voss.

**Writing – review & editing:** Nikolai Ramadanov, Robert Hable, Roland Becker, Andreas G. Schreyer, Robert Prill, Mikhail Salzmann.
